# Web-Based Mindfulness Interventions for People With Physical Health Conditions: Systematic Review

**DOI:** 10.2196/jmir.7487

**Published:** 2017-08-31

**Authors:** Kirsti I Toivonen, Kristin Zernicke, Linda E Carlson

**Affiliations:** ^1^ Department of Psychology University of Calgary Calgary, AB Canada; ^2^ Shared Mental Health Care Alberta Health Services Sheldon M. Shumir Health Centre Calgary, AB Canada; ^3^ Department of Psychosocial Resources Holy Cross Site Tom Baker Cancer Centre Calgary, AB Canada; ^4^ Department of Oncology University of Calgary Calgary, AB Canada

**Keywords:** Internet, mindfulness, review

## Abstract

**Background:**

Mindfulness-based interventions (MBIs) are becoming increasingly popular for helping people with physical health conditions. Expanding from traditional face-to-face program delivery, there is growing interest in Web-based application of MBIs, though Web-based MBIs for people with physical health conditions specifically have not been thoroughly reviewed to date.

**Objective:**

The objective of this paper was to review Web-based MBIs for people with physical health conditions and to examine all outcomes reported (eg, efficacy or effectiveness for physical changes or psychological changes; feasibility).

**Methods:**

Databases PubMed, PsycINFO, Science Direct, CINAHL Plus, and Web of Science were searched. Full-text English papers that described any Web-based MBI, examining any outcome, for people with chronic physical health conditions were included. Randomized, nonrandomized, controlled, and uncontrolled trials were all included. Extracted data included intervention characteristics, population characteristics, outcomes, and quality indicators. Intervention characteristics (eg, synchronicity and guidance) were examined as potential factors related to study outcomes.

**Results:**

Of 435 publications screened, 19 published papers describing 16 studies were included. They examined Web-based MBIs for people with cancer, chronic pain or fibromyalgia, irritable bowel syndrome (IBS), epilepsy, heart disease, tinnitus, and acquired brain injury. Overall, most studies reported positive effects of Web-based MBIs compared with usual care on a variety of outcomes including pain acceptance, coping measures, and depressive symptoms. There were mixed results regarding the effectiveness of Web-based MBIs compared with active control treatment conditions such as cognitive behavioral therapy. Condition-specific symptoms (eg, cancer-related fatigue and IBS symptoms) targeted by treatment had the largest effect size improvements following MBIs. Results are inconclusive regarding physical variables.

**Conclusions:**

Preliminary evidence suggests that Web-based MBIs may be helpful in alleviating symptom burden that those with physical health conditions can experience, particularly when interventions are tailored for specific symptoms. There was no evidence of differences between synchronous versus asynchronous or facilitated versus self-directed Web-based MBIs. Future investigations of Web-based MBIs should evaluate the effects of program adherence, effects on mindfulness levels, and whether synchronous or asynchronous, or facilitated or self-directed interventions elicit greater improvements.

## Introduction

### Mindfulness-Based Interventions

Given the increased public interest and research in both mindfulness-based interventions (MBIs) [1] and Internet-delivered therapies [2], this paper summarizes the research on Web-based MBIs for people with physical health conditions. Mindfulness practice involves moment-to-moment nonjudgmental awareness, applied by purposely attending to one’s own thoughts and bodily sensations with attitudes of openness and acceptance [3,4]. Although meditation practice originates from centuries of Buddhist tradition, it is often incorporated into structured, secular MBIs that are applied to a variety of clinical populations [1]. Several systematic reviews and meta-analyses have examined the effects of face-to-face group MBIs in heterogeneous clinical populations [5-8] as well as specific medical populations, including patients with chronic pain [9], fibromyalgia [10], multiple sclerosis [11], vascular disease [12], human immunodeficiency virus (HIV) and acquired immunodeficiency syndrome (AIDS) [13], and cancer [14-18]. These reviews have consistently described reduced anxiety, depressive symptoms, and distress; improved mood; and improved quality of life [5-7,11,12,14,16-18]. Theoretically, improvements in psychological well-being are driven by improved emotion regulation abilities, which in turn result in decreased rumination about the past, worry about the future, and experiential avoidance of difficult feelings (L Labelle, unpublished data, 2012). Ultimately, mindfulness practice can allow people to view their illness from a new perspective and result in the improvements that have been observed across a broad range of psychological and physical outcomes [8].

### Web-Based Interventions

There has been a surge in Web-based delivery of therapeutic interventions because of factors such as increased acceptability of the Internet as a social tool and continuous improvement in computer hardware and software (particularly concerning ease of use, privacy protection, and facilitating communication) [19]. Web-based therapies delivered through real time such as instant messaging platforms, telephone, or videoconferencing are categorized as *synchronous*, whereas delayed delivery methods such as email or message boards are categorized as *asynchronous*. Several reasons underlie the appeal of both types of Web-based therapies, including (1) ease and speed of accessibility by reducing wait-list times, (2) convenience of 24-hour availability for individual schedules (particularly with asynchronous therapies), (3) the ability for participants to work at their own pace in the comfort of their own homes, (4) allowing anonymity, and (5) reduced cost [20,21]. For those with physical health conditions in particular, home delivery can make participation in an intervention possible when it otherwise would not have been. For example, on-site attendance can be a barrier for those who experience limitations in mobility and energy levels or those who may have lower immunity to contagious diseases because of treatments such as chemotherapy and are avoiding groups of people. Additionally, the flexibility of Web-based delivery can be appealing for people who are busy managing appointments and treatment.

Whereas there is theoretical rationale for the utility of Web-based delivery of various therapies, and there have been hundreds of randomized controlled trials (RCTs) evaluating Internet-delivered health interventions [2], it remains unclear as to what extent people with physical health conditions currently use Web-based interventions, or Web-based MBIs specifically. Further complicating the question of use are the numerous mindfulness-based mobile apps that are widely available but have not been studied [22]. Although the actual uptake of Web-based therapies in this population is unknown, results from a recent Web-based survey suggest that people may prefer Internet interventions to in-person therapy. Wahbeh and colleagues [23] surveyed 500 people with a high prevalence of posttraumatic (70.6%, 353/500) and depressive (76.2%, 381/500) symptoms from the United States regarding their preferred delivery format of a mindfulness meditation intervention. Most (71.2%, 356/500) expressed interest in a Web-based format, and Internet delivery was the first choice of format for the greatest proportion of participants (42.7%, 212/496), followed by individual (37.8%, 187/496) and group (19.6%, 97/496). As participants were members of the general public and not people suffering from any specific medical or psychiatric conditions, the generalizability of findings to these groups is likely to be high but not certain. Despite the appeal of Web-based therapies, some potential drawbacks necessitate consideration. First, Web-based delivery may offer less interpersonal interaction and social support than in-person delivery. Second, compliance may be more difficult to determine if those delivering the intervention cannot directly observe those receiving it. Third, particularly with asynchronous interventions, it could take longer for participants to receive feedback and have questions answered. Fourth, monitoring of participants for adverse reactions may be more difficult in the Web-based environment, when personal contact may be absent. Referrals to appropriate mental health or medical services for any identified adverse reactions or need for further individualized treatment may also be more difficult in the Web-based environment where providers may be very geographically distant from patients. Finally, ethical issues around responsibility for care may also arise [24]. Thus, the benefits and drawbacks of Web-based therapies as well as treatment preferences should be considered when devising treatment plans. Ultimately, if effective, Web-based MBIs can be a more practical option for many people with physical health conditions and may remove barriers that would have otherwise precluded participation in face-to-face interventions.

### Prior Reviews of Web-Based MBIs

Given the popularity of Internet interventions and their potential for widespread application, it is important to evaluate their effectiveness as they are being developed and disseminated. A 2016 review and meta-analysis by Spijkerman et al [25] examined 15 RCTs of Web-based MBIs for improving mental health in a heterogeneous sample, including studies of healthy adults, students, and employees (*n*=7); people with psychiatric disorders or symptoms (*n*=3); and people with chronic pain or other physical illnesses (*n*=5). Of the 15 interventions, nine were guided (including real-time group or individual sessions or individual email correspondence) and six were unguided. Only three interventions were delivered as a virtual classroom, whereas 12 were delivered through websites or mobile phone apps. Most studies (*n*=10) compared treatment groups with wait-list controls. Five studies compared a treatment group with an online discussion forum, psychoeducation, or behavioral activation. Interventions typically comprised weekly sessions and were 2 to 12 weeks in duration. Web-based MBIs resulted in small effect size improvements in depression (Hedge *g*=0.29), anxiety (*g*=0.22), well-being (*g*=0.23), and mindfulness (*g*=0.32), as well as moderate effect size decreases in stress (*g*=0.51). Exploratory subgroup analyses showed that Web-based MBIs with therapist guidance resulted in greater effect size improvements in stress and mindfulness than those without guidance. The authors concluded that Web-based MBIs could be beneficial for mental health outcomes, particularly stress [25].

This review expands upon the review by Spijkerman et al, focusing on chronic physical health conditions alone and looking at both mental and physical health outcomes. Populations with primary psychological disorders were excluded from this review to prevent redundancy with Spijkerman et al [25], which focused on mental health. Furthermore, a narrower focus on populations presenting primarily with physical health conditions was selected because examination of a relatively homogeneous population may lead to more cohesive results specific to these groups.

## Methods

This review was conducted in accordance with the 2009 Preferred Reporting Items for Systematic Reviews and Meta-Analyses (PRISMA) guidelines for systematic reviews [26]. The literature search, paper screening (title and abstract, and full-text), and data extraction were undertaken by KT.

### Eligibility Criteria

Inclusion criteria were studies examining individuals with chronic physical conditions that received a Web-based MBI. All types of physical health conditions were included. Studies of acceptance and commitment therapy (ACT) and dialectical behavior therapy were included as MBIs because they contain elements of mindfulness practice (eg, sustaining attention in the present and nonjudgmental observation of emotion), though they involve less formal mindfulness meditation training than other structured MBIs [27]. RCTs, non-RCTs, and uncontrolled trials were included; and all outcomes reported by studies were described. Studies that described qualitative research or program evaluation, described populations with psychiatric disorders, did not have full-text in English available, or described interventions that included a small component of mindfulness but did not emphasize it (eg, multiweek programs where mindfulness practice is addressed only in one or two sessions) were excluded.

### Search Strategy

**Figure 1:**
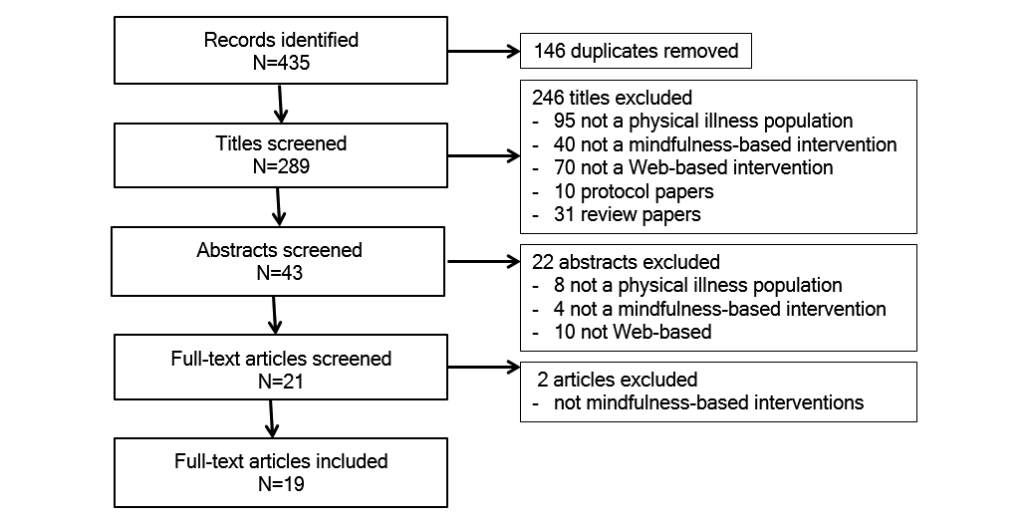
Study selection and inclusion flowchart.

A literature search was conducted in PubMed, PsycINFO, Science Direct, CINAHL Plus with Full Text, and Web of Science in the fields all fields, all fields, keywords, abstract, and topic, respectively. All searches used the terms “online OR Internet AND mindfulness AND intervention” (see Multimedia Appendix 1 for an example of the search strategy for PubMed). All papers published before and during November 2016 were included. Reference lists of included studies were searched to identify additional studies. Titles and abstracts and full-texts were screened simultaneously, such that when a paper may have met inclusion criteria based on the title and abstract screen, the full-text was immediately screened. See Figure 1 for a flowchart of study selection and inclusion.

### Data Extraction

For each study, the following data were extracted: first author, year of publication, country, population characteristic (physical health condition, age, and sex), number of participants per group, study design (eg, randomized and nonrandomized), intervention characteristics (type of intervention, synchronous or asynchronous, delivery mode [eg, Web-based and videoconference], guided or unguided [where any correspondence with a therapist, including email, was considered guided], number of sessions, and duration), assessment times (pre, post, follow-up), all outcomes (see Table 1), and adherence and dropout (see Table 2). Note that reporting of adherence and dropout was inconsistent across studies—it is described in this review as it was reported in the original studies. Data were extracted by KT according to a predetermined form.

Methodological quality was assessed based on potential sources of bias outlined in the Cochrane Handbook for Systematic Reviews of Interventions [28]. Sources of bias that were assessed at the study level included random sequence generation (ie, whether participants were randomly assigned to groups), allocation concealment (ie, whether group assignment was unknown when participants were recruited), blinding of participants and personnel (ie, whether participants and people involved in the study were unaware of participant group assignment), and blinding of outcome assessment (ie, whether the individual rating the outcome measure was aware of group assignment). Sources of bias assessed at the outcome level were complete outcome data or intention-to-treat (ITT) analysis used (where outcome data were considered complete if ≥90% of those randomized were included), and whether all the outcomes that were described in the Methods section were reported. Note that studies were coded as yes when they met the criteria and no when they either did not meet criteria or when it was ambiguous as to whether they met the criteria.

## Results

Overall, 19 published papers describing 16 studies of Web-based MBIs for physical health conditions were identified through the literature search and are subsequently described [29-47]. Three of the included papers describe long-term, follow-up results or secondary analyses that were separately published [32,37,46]. Thus, whereas all 19 papers are described in text where relevant, only the original 16 are presented in Tables 1-3 and described subsequently. Interventions with the greatest emphasis on mindfulness included Mindfulness-Based Stress Reduction (MBSR; n=1) [44], Mindfulness-Based Cancer Recovery (MBCR; n=1) [45], Mindfulness-Based Cognitive Therapy (MBCT; n=4) [30,41,42,47], Mindful Socioemotional Regulation (MSER; n=1) [33], Mindfulness-Based Chronic Pain Management (MBCPM; n=1) [34], or general mindfulness training (n=1) [35]. Interventions that included mindfulness as a component of a multimodal intervention were ACT (n=3) [29,31,43] and cognitive behavioral therapy (CBT) plus mindfulness (n=4) [36,38-40]. Most studies employed a randomized controlled design (n=13) and nearly all (n=15) had at least one comparison group. Seven studies had an active comparison: the same MBI delivered in person (n=2) [34,44], a control group receiving another Web-based therapy (n=4) [31,38,40,43], or a walking control group (n=1) [44]. The remaining studies had a psychoeducation control (n=2) [30,33], an online discussion forum control (n=4) [29,36,39,43], or wait-list or treatment as usual (n=6) [31,34,35,41,42,45]. Note that four studies had two comparison groups [31,34,43,44]. Most interventions included a form of guidance: either self-facilitated interventions including email correspondence with a therapist (n=8) [29,31,36,38-40,43,47] or therapist-facilitated sessions (n=5) [34,41,42,44,45]. Five studies were conducted with populations with chronic pain [29-31,34] or fibromyalgia [33] (which has been grouped with chronic pain in this review, as it is characterized by widespread chronic pain). Other populations examined included people with heart disease (n=1) [35], irritable bowel syndrome (IBS; n=4) [36,38-40], epilepsy (n=2) [41,42], tinnitus (n=1) [43], acquired brain injury (n=1) [44], and cancer (n=2) [45,47]. The largest number of studies were conducted in Sweden (n=7) [29,36,38-40,43,44], followed by the United States (n=3) [33,41,42], the Netherlands (n=3) [31,35,47], Canada (n=2) [34,45], and Ireland (n=1) [30]. See Table 1 for study characteristics.

Regarding primary outcomes, five studies assessed pain or related constructs (eg, pain acceptance and pain interference) [29-31,33,34], four studies assessed IBS symptoms or symptom severity [36,38-40], two assessed depressive symptoms [41,42], two assessed fatigue [44,47], one assessed exercise capacity [35], one assessed tinnitus distress severity [43], and one assessed feasibility [45]. Four studies [30,33,34,36] assessed more than one primary outcome, including outcomes such as affect, social measures, and health-related quality of life (HRQoL). Table 1 outlines a complete list of primary and secondary outcomes assessed per study. More than half (*n*=9) of the studies included follow-up assessments [29-31,36,38-40,42,43] that ranged from 3 months [36] to 12 months post intervention [39,43]. See Table 2 for summary of primary outcomes, including the mean number of sessions completed, whether primary outcomes improved over time and relative to control, and whether improvements were maintained at follow-up, where applicable. Note that if a study had both an active and wait-list control, Table 2 presents improvement over the wait-list control.

**Table 1:** Summary of study characteristics.

First author (year)	Population, country	Mean age in years (% F)^a^	Intervention type	Number of sessions, duration	Control condition	Outcomes^b^
Gardner-Nix (2008) [34]	Chronic pain, Canada	51-54 (75-88)	Synchronous videoconference MBCPM^c^, guided (n=57)	10 sessions, 10 weeks	On-site program (n=99), wait-list (n=59)	*Health-related quality of life (physical and mental), pain catastrophizing, pain*
Buhrman (2013) [29]	Chronic pain, Sweden	49 (59)	Asynchronous Web-based ACT^d^, guided (n=38)	7 sessions, 7 weeks	Online discussion forum (n=38)	*Pain acceptance*, anxiety and depressive symptoms, coping, pain consequences, relationship with pain, quality of life
Davis (2013) [33]	Fibromyalgia, United States	46 (98)	Asynchronous Web-based MSER^e^, unguided (n=39)	12 sessions, 6 weeks	Web-based health behavior information (n=40)	*Pain, pain coping efficacy, positive affect, negative affect, social engagement, loneliness, family stress, family enjoyment, stress coping efficacy*
Dowd (2015) [30]	Chronic pain, Ireland	45 (90)	Asynchronous Web-based MBCT^f^, unguided (n=62)	12 sessions, 6 weeks	Web-based psychoeducation (n=62)	*Pain interference, psychological distress*, pain intensity, catastrophizing, pain acceptance, mindfulness, life satisfaction, impression of change
Trompetter (2015) [31]	Chronic pain, the Netherlands	52-53 (75-77)	Asynchronous Web-based ACT^d^, guided (n=82)	9 modules, 9-12 weeks	Web-based expressive writing (n=79), wait-list (n=77)	*Pain interference*, anxiety and depressive symptoms, pain intensity, pain disability, positive mental health, psychological inflexibility, mindfulness, engaged living, pain catastrophizing
Younge (2015) [35]	Heart disease, the Netherlands	43 (44-51)	Asynchronous Web-based mindfulness training, unguided (n=215)	4 parts, 12 weeks	TAU^g^ (n=109)	*Exercise capacity*, heart rate, blood pressure, respiration rate, NT-proBNP^h^, health status, perceived stress, psychological distress, social support, and composite “endpoint” score
Ljótsson (2010) [36]	IBS^i^, Sweden	35 (85)	Asynchronous Web-based mindfulness and exposure CBT^j^, guided (n=42)	5 steps, 10 weeks	Online discussion forum (n=43)	*IBS*^i^*symptoms and symptom severity*, quality of life, gastrointestinal-specific anxiety, depression, disability, treatment credibility
Ljótsson (2011) [38]	IBS^i^, Sweden	38 (79)	Asynchronous Web-based mindfulness and exposure CBT^j^, guided (n=98)	5 steps, 10 weeks	Web-based stress management program (n=97)	*IBS*^i^*symptom severity*, quality of life, cognitive symptoms for bowel disorders, perceived stress, anxiety and depression, symptom relief
Ljótsson (2011) [39]	IBS^i^, Sweden	35 (74)	Asynchronous Web-based mindfulness and exposure CBT^j^, guided (n=30)	5 steps, 10 weeks	Online discussion forum (n=31)	*IBS*^i^*symptom severity*, health economic data, quality of life, gastrointestinal-specific anxiety, disability
Ljótsson (2014) [40]	IBS^i^, Sweden	42 (80)	Asynchronous Web-based mindfulness and exposure CBT^j^, guided (n=153)	5 steps, 10 weeks	Same mindfulness CBT^j^ without exposure (n=156)	*IBS*^i^*symptom severity*, quality of life, gastrointestinal-specific anxiety, cognitive symptoms for bowel disorders, anxiety and depressive symptoms
Thompson (2010) [41]	Epilepsy, United States	36 (81)	Synchronous MBCT^f^, phone-based or Web-based, guided (n=26)	8 sessions, 8 weeks	TAU^g^ wait-list (n=27)	*Depressive symptoms*, knowledge and skills, self-efficacy, satisfaction with life, quality of life, self-compassion
Thompson (2015) [42]	Epilepsy, United States	41 (65)	Synchronous MBCT^f^, phone-based or Web-based, guided (n=64)	8 sessions, 8 weeks	TAU^g^ wait-list (n=64)	*Depressive symptoms*, knowledge and skills, self-efficacy, satisfaction with life, quality of life, self-compassion
Hesser (2012) [43]	Tinnitus, Sweden	49 (43)	Asynchronous Web-based ACT^d^, guided (n=35)	8 sessions, 8 weeks	CBT^j^ (n=32), online discussion forum (n=32)	*Tinnitus distress severity*, anxiety and depressive symptoms, insomnia severity symptoms, perceived stress, quality of life
Johansson (2015) [44]	Acquired brain injury, Sweden	46-51 (82)	Synchronous Web-based MBSR^k^, guided (n=13)	8 sessions plus 7-hr retreat, 8 weeks	On-site MBSR^k^ (n=12), group walking control (n=9)	*Mental fatigue*, anxiety and depressive symptoms, self-compassion, measures of attention and processing speed
Zernicke (2014) [45]	Cancer, Canada	58 (73)	Synchronous Web-based MBCR^l^, guided (n=30)	8 sessions plus 6-hr online retreat, 8 weeks	TAU^g^ wait-list (n=32)	*Feasibility*, mood, stress, posttraumatic growth, spirituality and well-being, mindfulness
Bruggeman-Everts (2015) [47]	Cancer, the Netherlands	50 (76)	Asynchronous Web-based MBCT^f^, guided (n=257)	9 sessions, 9 weeks	None	*Fatigue severity*, psychological distress

^a^Mean age in years and % F (% female) are presented as ranges when original studies reported this information per group rather than for the whole sample.

^b^Primary outcomes italicized.

^c^MBCPM: Mindfulness-Based Chronic Pain Management.

^d^ACT: acceptance and commitment therapy.

^e^MSER: Mindful Socioemotional Regulation.

^f^MBCT: Mindfulness-Based Cognitive Therapy.

^g^TAU: treatment as usual.

^h^NT-proBNP: N-terminal probrain natriuretic peptide.

^i^IBS: irritable bowel syndrome.

^j^CBT: cognitive behavioral therapy.

^k^MBSR: Mindfulness-Based Stress Reduction.

^l^MBCR: Mindfulness-Based Cancer Recovery.

**Table 2:** Summary of outcomes.

First author (year)	Sample diagnosis	Mean sessions completed, %^a^	Primary outcome	Improvement^b^		
				Over time	Greater than comparison	Maintained at follow-up
Gardner-Nix (2008) [34]	Chronic pain	Unclear	Quality of life—physical health	No	No	N/A^c^
			Quality of life—mental health	Yes	Yes^d^	N/A
			Pain catastrophizing	Yes	Yes^d^	N/A
			Usual pain	Yes	Yes^d^	N/A
Buhrman (2013) [29]	Chronic pain	60	Pain acceptance	Yes	Yes	Yes (6 mo^e^)
Davis (2013) [33]	Fibromyalgia	69	Pain	No	No	N/A
			Pain coping efficacy	Yes	Yes	N/A
			Positive affect	Yes	Yes	N/A
			Negative affect	Yes	No	N/A
			Social engagement	Yes	Yes	N/A
			Loneliness	Yes	Yes	N/A
			Family stress	Yes	No	N/A
			Family enjoyment	Yes	Yes	N/A
			Stress coping efficacy	Yes	Yes	N/A
Dowd (2015) [30]	Chronic pain	94^f^	Pain interference	Yes	No	Yes (6 mo)
			Psychological distress	No	No	No (6 mo)
Trompetter (2015) [31]	Chronic pain	Unclear	Pain interference	Yes	No^g^	No^g^ (6 mo)
Younge (2015) [35]	Heart disease	Unclear	Exercise capacity	Yes	Marginally (*P*=.05)	N/A
Ljótsson (2010) [36]	IBS^h^	Unclear	IBS symptoms	Yes	Yes	N/A
			IBS symptom severity	Yes	Yes	Yes (3 mo)
Ljótsson (2011) [38]	IBS	Unclear	IBS symptom severity	Yes	Yes	Yes (6 mo)
Ljótsson (2011) [39]	IBS	Unclear	IBS symptom severity	Yes	Yes	Yes (12 mo)
Ljótsson (2014) [40]	IBS	Unclear	IBS symptom severity	Yes	Yes	Yes (6 mo)
Thompson (2010) [41]	Epilepsy	75^i^	Depressive symptoms	Yes	Yes	N/A
Thompson (2015) [42]	Epilepsy	83	Depressive symptoms	Yes	Yes	Yes (4.5-5 mo)
Hesser (2012) [43]	Tinnitus	Unclear	Tinnitus distress severity	Yes	Yes^d^	Yes (12 mo)
Johansson (2015) [44]	Acquired brain injury	Unclear	Mental fatigue	Yes	Yes	N/A
Zernicke (2014) [45]	Cancer	67	Feasibility	N/A	N/A	N/A
Bruggeman-Everts (2015) [47]	Cancer	70	Fatigue severity	Yes	N/A	N/A

^a^Mean intervention completion refers to the mean % of modules the participants participated in.

^b^Improvement—Over time and Improvement—Greater than comparison refer to postintervention assessments.

^c^N/A: not applicable.

^d^Greater than wait-list control but not greater than on-site comparison group.

^e^mo: months.

^f^Of those who completed follow-up.

^g^Greater than active comparison group but not wait-list control.

^h^IBS: irritable bowel syndrome.

^i^Median reported.

### Chronic Pain and Fibromyalgia

Five of the identified studies examined a Web-based MBI for populations with chronic pain or fibromyalgia, an illness characterized by chronic pain. Of these, four studies were RCTs [29-31,33], whereas one study was not randomized [34]. Gardner-Nix and colleagues [34] compared MBCPM delivered via videoconference or on-site to wait-list control in chronic pain patients. Most participants (70%, 40/57) remained in the videoconference group. Physical HRQoL improved more in the on-site group, and distance delivery was no better than control, whereas mental HRQoL improved in both on-site and distance groups relative to control. Pain catastrophizing improved in both on-site and distance groups relative to control groups, and actual pain improved more in the distance group than the control group but more in the on-site group than the distance group.

Burhman and colleagues [29] compared an asynchronous Web-based ACT program with a wait-list control with an online discussion group for 76 patients with chronic pain. Less than half (40%, 15/38) of the treatment group completed all seven sections. The primary outcome, chronic pain acceptance, was higher in the treatment group than control (*d*=0.41) and did not change at a 6-month follow-up. Of secondary outcomes, anxiety and depressive symptoms improved more in the treatment group than the control (*d*=0.44 and *d*=0.18, respectively) and did not change at a 6-month follow-up. Two of eight subscales on a measure of coping (*d*=0.28-0.51) and two of eight subscales on a measure of pain symptoms (*d*=0.30-0.56) improved more in the treatment group and were maintained at follow-up. There were no effects on quality of life, pain impairment, or relationship with pain.

Davis and Zautra [33] compared Web-based MSER with a Web-based health education attention control group in an RCT of 79 fibromyalgia patients. Approximately half (49%, 19/39) completed all 12 MSER modules. Those who participated in MSER experienced greater improvement in pain coping efficacy, positive affect, social activity engagement, loneliness, family enjoyment, and stress coping relative to control (effect sizes, representing within-group variance accounted for by group assignment, ranged from .01 to .06). Both groups resulted in decreased negative affect, and neither group experienced decreased pain symptoms.

Dowd and colleagues [30] compared Web-based MBCT with Web-based pain management psychoeducation for adults (n=124) with chronic pain. Whereas 45% (28/62) and 37% (23/62) participants were retained at post and follow-up assessments, respectively, most of those who completed the follow-up assessment (74%, 17/23) viewed all sessions. Additionally, 6-month follow-up measures were included in the primary analyses. Of the primary outcomes, pain interference improved across both groups (*d*=0.76), but psychological distress did not improve for either group. Of secondary outcomes measured, satisfaction with life improved more in the mindfulness group than psychoeducation (*d*=0.59); pain acceptance, mindfulness, and catastrophizing all improved in both groups over time (*d*=0.42-0.58). No significant improvements were observed in average pain experienced, and a trend toward less “pain right now” across both groups was observed. Impression of change was measured with three subscales (ability to manage emotions, dealing with stressful situations, ability to enjoy pleasant events)—all of which improved more in the treatment group (*d*=0.41-0.62) and were maintained at 6 months, except for ability to enjoy pleasant events, which improved post intervention but was not maintained.

Trompetter and colleagues [31] compared Web-based ACT with an expressive writing control or wait-list control group for 238 people with chronic pain. Nearly half (48%, 39/82) adhered to ACT according to the author’s definition of participating for ≥3 hours per week. The primary outcome, pain interference, was more improved in the ACT group than the expressive writing group at the 3-month (post intervention; *d*=0.33) and 6-month follow-ups (*d*=0.47), but not the wait-list group. Regarding secondary outcomes, at post treatment, ACT outperformed expressive writing for improving pain intensity, outperformed wait-list for improving pain catastrophizing, and outperformed both groups for improving psychological inflexibility (*d*=0.23-0.60). At the 6-month follow-up, ACT outperformed expressive writing for improving pain disability, outperformed wait-list for improving mindfulness, and outperformed both groups for improving depressive symptoms, pain intensity, psychological inflexibility, and catastrophizing (*d*=0.28-0.54). No differences were observed between groups on anxiety, positive mental health, or engaged living outcomes. In follow-up analyses of their results, Trompetter and colleagues concluded that changes in psychological flexibility mediated the observed changes in pain interference, psychological distress, and pain intensity and suggested that pain catastrophizing served as an indirect mechanism of change through its effect on psychological flexibility [32].

Taken together, results support improvements in psychological outcomes and pain coping efficacy following Web-based MBIs for populations experiencing chronic pain but are mixed regarding the effectiveness of MBIs for decreasing actual symptoms of pain. Specifically, two studies reported improved pain over control groups, one study reported a trend toward pain improvement across treatment and control groups, and two reported no improvement in pain symptoms. As only one of the studies examining pain was synchronous [34], no conclusions can be drawn regarding the relative effectiveness of synchronous or asynchronous interventions for people with chronic pain. There appeared to be no difference between guided or unguided interventions. Only one study [34] compared a Web-based MBI to the same intervention delivered in person. Although its results suggest that the Web-based MBI may be just as effective as an on-site MBI for outcomes such as pain catastrophizing and mental HRQoL, the study included methodological issues such as lack of randomization or allocation concealment (see Table 3).

**Table 3:** Risk of bias assessment. Outcome data are considered complete if ≥90% of those randomized were included in outcome data.

First author (year)	Random sequence generation	Allocation concealment	Blinding of participants and personnel	Blinding of outcome assessment	Complete outcome data or intention-to-treat analysis used	All outcomes reported
Gardner-Nix (2008) [34]	No	No	No	No	No	Yes
Buhrman (2013) [29]	Yes	Yes	No	No	Yes	Yes
Davis (2013) [33]	Yes	Yes	No	No	Yes	Yes
Dowd (2015) [30]	Yes	Yes	No	No	Yes	Yes
Trompetter (2015) [31]	Yes	Unclear	No	No	Yes	Yes
Younge (2015) [35]	Yes	Yes	No	Yes	No	No
Ljótsson (2010) [36]	Yes	Yes	No	No	Yes	Yes
Ljótsson (2011) [38]	Yes	Yes	No	Unclear	Yes	Yes
Ljótsson (2011) [39]	Yes	No	No	No	Yes	Yes
Ljótsson (2014) [40]	Yes	Yes	No	Unclear	Yes	Yes
Thompson (2010) [41]	Yes	Yes	No	No	No	Yes
Thompson (2015) [42]	Yes	Yes	No	No	Yes	Yes
Hesser (2012) [43]	Yes	Yes	No	No	Yes	Yes
Johansson (2015) [44]	No	No	No	No	No^a^	Yes
Zernicke (2014) [45]	Yes	Yes	No	No	Yes	Yes
Bruggeman-Everts (2015) [47]	No	No	No	No	Yes	Yes

^a^Intention-to-treat analysis conducted with a subset of participants but not all.

### Heart Disease

One large RCT, conducted by Younge and colleagues [35], compared unguided, Web-based mindfulness training with usual care in 324 patients with heart disease. The completion rate (defined by the authors as completing at least 50% of the intervention) was 53.5% (115/215) of those who were randomized to intervention. On the primary outcome, a measure of exercise tolerance (the 6-min walk test), the mindfulness group performed better than control at a difference bordering significance, and the mindfulness group showed lower resting heart rate (*d*=0.20). There were no differences between the groups on blood pressure, blood levels of N-terminal probrain natriuretic peptide (NT-proBNP), mental or physical HRQoL, subjective health status, anxiety and depressive symptoms, perceived stress, perceived social support, and adverse events (all-cause mortality, heart failure, symptomatic arrhythmia, cardiac surgery, or percutaneous cardiac intervention). An as-treated analysis showed small effect size improvements in exercise tolerance, heart rate, systolic blood pressure, and stress (*d*=0.19-0.21). Changes in weight or blood levels of creatinine were not reported. On the basis of this one large trial the intervention appears only marginally better than usual care.

### Irritable Bowel Syndrome

Ljótsson and colleagues conducted a series of RCTs examining Web-based mindfulness plus exposure-based CBT for people with IBS. Described in Ljótsson et al [36], the intervention was tested in 85 people with IBS randomized to the intervention or an online discussion forum control group. Most participants (69%, 29/42) in the intervention group completed all modules. The intervention group experienced large effect size reductions in primary outcome measures, a composite score of IBS symptoms (*d*=1.19) and IBS symptom severity (*d*=1.21) relative to the control group. All secondary outcome measures, IBS-related quality of life, gastrointestinal-specific anxiety, depressive symptoms, and perceived disability, improved more in the intervention group than control (*d*=0.43-0.93). IBS quality of life continued to improve at a 3-month follow-up for the intervention group, and IBS symptom severity and gastrointestinal-specific anxiety did not change. Long-term, follow-up results 15 to 18 months following treatment, after the wait-list group also received the treatment, showed that the improvements in IBS symptom severity, IBS quality of life and gastrointestinal-related anxiety were maintained [37].

Ljótsson et al [38] then tested the same Web-based mindfulness plus CBT treatment in an RCT against an active control—a Web-based stress management group [38]. The primary outcome, IBS symptom severity, was more improved in the intervention group than the active control (*d*=0.38). Primary analyses included pre, post, and 6-month follow-up assessments. Improvements in the intervention group relative to control were observed for secondary outcomes IBS quality of life, gastrointestinal-related anxiety, and an IBS-related cognitions scale (including negative thoughts about bowel function and personality characteristics thought to be linked to IBS; *d*=0.33-0.52). Perceived stress and anxiety and depressive symptoms improved over time for both conditions. At post, those in the intervention group did not feel significantly more symptom relief than control (69%, 68/98, vs 58%, 56/97) but at a 6-month follow-up the difference was significant (65%, 64/98, vs 44%, 43/97).

Ljótsson et al [39] conducted another study comparing Web-based mindfulness plus CBT with a wait-list online discussion forum for IBS patients recruited from a gastrointestinal clinic. The primary outcome, IBS symptom severity, had greater reductions in the intervention group relative to control (*d*=0.77). All secondary outcomes—IBS-related quality of life, gastrointestinal-specific anxiety, and perceived disability—improved more in the intervention group relative to the control group (*d*=0.19-0.79). Scores for IBS quality of life further improved and all other outcomes were maintained at a 12-month follow-up in the intervention group.

Most recently, Ljótsson et al [40] investigated whether the systematic exposure component had incremental effects over the other components of the mindfulness plus CBT therapy in a randomized controlled dismantling design. Participants with IBS (n=309) received the usual intervention or a version of it without the systematic exposure component. The primary outcome, IBS symptom severity, improved more in the intervention group with exposure post treatment (*d*=0.47) and was maintained at a 6-month follow-up. Other secondary outcomes (gastrointestinal-specific anxiety, IBS-related quality of life, and anxiety and depressive symptoms) improved more in the group with exposure (*d*=0.18-0.36). There was no difference regarding cognitions related to IBS between the two groups. The authors concluded that systematic exposure had incremental benefits over the other components of the intervention. In sum, this line of research has consistently demonstrated the beneficial effects of a Web-based mindfulness plus CBT treatment for IBS symptoms, quality of life, and psychological distress among people with IBS. Although it remains unclear as to what extent therapist guidance contributed to the positive results, results suggest that the active exposure component is an important implicated process.

### Epilepsy

Thompson and colleagues conducted two RCTs to examine the effectiveness of a distance delivery version of MBCT for depressive symptoms in people with epilepsy. First, in 2010, they randomly assigned 53 people with epilepsy to receive distance MBCT (via the Internet or telephone) or a wait-list control in a stratified cross-over design [41]. The primary outcome, depressive symptoms, improved more in the intervention group than control. Whereas results were slightly better for the phone group, there was no significant difference between telephone or Internet delivery. Regarding secondary outcomes, knowledge and skills (about epilepsy and depression) improved more in the MBCT group compared with control, but no significant differences between groups were observed for the outcomes self-efficacy, satisfaction with life, or quality of life. Once all individuals received the intervention, only 30% (13/44) participated in every session. Effect sizes were not reported.

Most recently, Thompson et al [42] examined distance-delivered MBCT for the prevention of depressive symptoms in people with epilepsy. Participants (n=128) were randomized to the same MBCT intervention or a wait-list condition. The primary outcome, depressive symptoms, improved more in the treatment group than control and remained lower than baseline at an 18 to 20 week follow-up. There was reduced incidence of depressive episodes in the treatment group relative to control. Regarding the secondary outcomes, knowledge and skills and satisfaction with life both improved more in the treatment group than control. Effect sizes were not reported. There were no significant changes in the treatment group over control in the outcomes depression coping self-efficacy, self-compassion, or quality of life.

Considered together, results suggest that Web-based MBCT is effective for reducing depressive symptoms among patients with epilepsy as well as for educating them about depression in the context of their illness but does not improve self-efficacy or quality of life. As the Web-based MBCT delivered in both studies was guided, synchronous and compared with treatment as usual, it is difficult to determine to what extent the intervention accounted for the observed changes in depressive symptoms, or which other factors (eg, receipt of treatment in general and social support) may have contributed.

### Tinnitus

Hesser and colleagues [43] conducted an RCT comparing ACT with CBT and an online discussion forum control condition for people with tinnitus experiencing significant distress (n=99). The primary outcome, tinnitus distress severity, improved more in the ACT condition than control (*d*=0.68). This improvement did not differ between ACT and CBT conditions and was maintained at a 1-year follow-up. Regarding secondary outcomes, depressive symptoms, anxiety, and perceived stress all improved more in the ACT group than the control group (*d*=0.59-0.69). There were no differences between ACT and control on outcomes insomnia symptom severity and quality of life. Authors concluded that for coping with tinnitus, ACT may be as effective as CBT, which they noted was currently the most supported psychological intervention for tinnitus management. Data describing rates of module completion among those who started the study were not reported.

### Acquired Brain Injury

A small nonrandomized trial compared Web-based MBSR with a control condition that comprised weekly walking sessions for those with acquired brain injuries (either from traumatic brain injury or stroke) experiencing mental fatigue (n=34) [44]. Of the outcomes examined, only mental fatigue reduced significantly more in the Web-based MBSR condition than both the face-to-face and walking control conditions. Depression, anxiety, and attention and processing speed measures improved within the Web-based MBSR group, whereas self-compassion did not change. Mental fatigue also significantly decreased for the walking control group when they were subsequently given Web-based MBSR. The mean attendance rates were 81% in the initial Web-based MBSR group, 82% when the walking control was subsequently given Web-based MBSR, and 94% in the face-to-face MBSR group. Authors concluded that Web-based MBSR can be helpful in improving mental fatigue, though this study included several methodological issues such as lack of randomization or allocation concealment (see Table 3).

### Cancer

Two published studies have examined Web-based MBIs for cancer survivors. Zernicke and colleagues [45] compared a Web-based synchronous MBCR program with wait-list control for cancer survivors (n=62) in an RCT. The program was considered feasible (the primary outcome) because of exceeded target numbers for interest and recruitment. Regarding secondary outcomes, participants in the MBCR group reported greater improvements in mood, stress symptoms, spirituality, and acting with awareness relative to the control group (*d*=0.37-0.50). Both groups reported improvements in posttraumatic growth, and in four subscales of a mindfulness measure (mindfulness observing, describing, nonreacting, and nonjudging). After the wait-list control group subsequently received the Web-based MBCR intervention, Zernicke et al [46] published exploratory analyses of outcomes and associations between outcomes and age, sex, and cancer stage. Mood and mindfulness subscales, awareness, observing, describing, and nonjudging, improved overall following the intervention. Stress symptoms, spirituality, and the mindfulness subscale nonreacting improved more among younger than older participants. Posttraumatic growth improved more for men than women, though men had significantly worse posttraumatic growth scores at baseline. There were no differential effects based on cancer stage.

In a large trial using a one-group pre-post design, Bruggeman-Everts and colleagues [47] examined the effectiveness of Web-based MBCT for improving cancer-related fatigue in 257 patients. The primary outcome, fatigue severity, significantly decreased following participation in the intervention (*d*=1.45). Regarding secondary outcomes, psychological distress decreased (*d*=0.71), clinically significant improvement in fatigue severity occurred in 35% (89/257) of patients, and most endorsed satisfaction with the intervention.

Taken together, these results suggest that Web-based MBIs for cancer survivors are effective for improving fatigue symptoms, mood, and psychological distress. It is unclear whether guidance is related to improved outcomes as both interventions included some form of guidance. It is also unclear whether synchronous or asynchronous interventions may be more effective for cancer survivors. As Zernicke et al [45] compared MBSR with a wait-list control group and Bruggeman-Everts et al [47] had no comparison group, it is also unclear whether Web-based MBIs would be effective for fatigue, mood, and distress outcomes beyond an active control condition.

Overall, of 28 primary outcomes (excluding feasibility) from the 16 studies, 25 of the primary outcomes improved over time, and 20 of 27 improved more in intervention groups than in control groups (one primary outcome was from a study that did not have a control group). Of the 10 primary outcomes assessed at follow-up, eight showed maintained improvements (see Table 2).

### Risk of Bias

Table 3 summarized the risk of bias assessment based on potential sources of bias outlined in the Cochrane Handbook for Systematic Reviews of Interventions [28]. Of the 16 studies, 15 reported all outcomes [29-31,33,34,36,38-45,47], 13 used random sequence generation [29-31,33,35,36,38-43,45], 11 had allocation concealment [29,30,33,35,36,38,40-43,47], and 12 had complete outcome data or used ITT analysis [29-31,33,36,38-40,42,43,45,47]. Only one study had blinded outcome assessments [35] and none blinded participants or personnel.

## Discussion

### Principal Findings

Nineteen published papers describing 16 studies examining Web-based MBIs for people with chronic physical health conditions were reviewed. Overall, most primary outcomes improved over time, but results were mixed as to whether they improved more in the intervention group relative to the control group. Specifically, outcomes including pain acceptance, stress coping efficacy, family enjoyment, social engagement, depressive symptoms, and fatigue all improved more after mindfulness interventions than control conditions, whereas other outcomes such as psychological distress, pain interference, and negative affect did not. Furthermore, among the studies including both active and wait-list control groups, some symptoms improved more in the Web-based MBI group than the wait-list control but not the active comparison group [34,43], and one study reported greater improvement in the Web-based MBI group than the active comparison but not the waitlist control [31]. Among all studies, anxiety and depressive symptoms were most frequently included as outcome variables and typically improved to a greater degree in mindfulness groups than control groups. This is consistent with reviews describing improvements in anxiety and depressive symptoms following participation in face-to-face MBIs for people with physical health conditions [5-7]. Evidence was mixed regarding the effectiveness of Web-based MBIs for improving the quality of life, but improvements in quality of life following interventions relative to control were reported more often than no improvements were reported. Although few studies examined mood, positive affect, stress, and social outcomes, improvements in these areas following intervention relative to control were reported. Among the few studies that examined distress, there was no evidence suggesting that MBIs caused improvement over a control condition. The mixed results for improvements and distress and quality of life are consistent with review by Goyal et al [5].

Whereas none of the studies included blinding of participants and personnel, this is not always possible in an RCT where a mindfulness intervention is being delivered. However, this criterion was not removed from the bias assessment in recognition that expectations surrounding the effectiveness of MBIs from participants and those who deliver interventions may represent sources of positive bias. Furthermore, lack of blinding of participants precluded blinded outcome assessment for many of these studies, as most outcomes were assessed with self-report measures. Beyond these inherent limitations, just over half (9/16) of the studies in Table 3 met all other quality indicators of randomization, allocation concealment, complete data or ITT analyses, and reporting all outcomes.

Some research groups showed great success implementing Web-based MBIs for specific populations. Ljótsson and colleagues investigated mindfulness plus CBT for IBS in a series of studies [36-40], showing consistent improvements in IBS symptom severity and quality of life in treatment groups relative to control groups. Thompson and colleagues [41,42] consistently demonstrated the effectiveness of Web-based MBIs for reducing depressive symptoms among those with epilepsy, and the effectiveness of MBCT for improving cancer-related fatigue reported by Bruggeman-Everts et al [47] is promising. It may follow that Web-based MBIs could be most effective when tailored and targeted for specific symptoms in specific populations. Indeed, the largest effect sizes of all studies reviewed were those reported for reduction of IBS symptom severity (*d*=1.21) [36] and cancer-related fatigue severity (*d*=1.45) [47], though the latter effect size represents the pre-post change for the intervention group only, not relative to a control, so it would not likely be as large in a controlled study. Both of these studies were tailored to address specific symptoms, included therapist guidance through correspondence, and included both mindfulness and CBT components. As the evidence for MBCT for cancer-related fatigue though would be more compelling if improvement was found to be greater relative to an active control group, this research group plans to investigate MBCT for cancer-related fatigue in a 3-armed RCT [48].

Younge et al [35] examined a variety of physical outcomes (eg, exercise tolerance, heart rate, blood pressure, and NT-proBNP), reporting only significant improvements in heart rate and marginally significant improvement in exercise tolerance. Although there is strong evidence to support reductions in blood pressure in cardiovascular disease populations following in-person MBIs [8], this was not replicated in the 2015 study by Younge and colleagues [35]. However, as there were a considerable number of dropouts from this study, further studies examining physical outcomes should be conducted before conclusions regarding the effectiveness of Web-based MBIs for objective physical outcomes such as blood pressure can be drawn. Whereas pain is not an objective physical outcome as it can only be ascertained by self-report, it can arguably be considered, at least in part, a physical outcome. In this review, there were mixed results regarding the effectiveness of Web-based MBIs for reducing pain specifically, but improvements occurred among outcomes reflecting abilities to regulate and deal with pain such as pain acceptance, catastrophizing, coping, and perceived pain-related disability. This is consistent with early work from Jon Kabat-Zinn et al [49] showing that people with chronic pain learned through MBSR to relate to their pain in a different and less distressing way, although the pain itself did not diminish significantly. Thus, whereas reductions in actual pain may not follow participation in Web-based MBIs per se, participants develop skills to regulate pain symptoms that may ultimately lead to less pain-related distress. Overall, the stronger evidence for impact on psychological than physical outcomes observed in this review is consistent with prior reviews of MBIs, which describe higher effect sizes for psychological outcomes than for physical outcomes [6].

The review of Web-based MBIs for improving mental health outcomes by Spijkerman et al [25] reports small effect size improvements for depression, anxiety, well-being, and mindfulness and moderate effect size for stress (though the authors cautioned that one outlier may inflate the effectiveness of MBIs for stress in their review). The authors concluded that the effect sizes they observed for psychological outcomes following Web-based MBIs were generally lower than the medium to large effect sizes found for the same outcomes in studies of face-to-face MBIs. They considered that the inclusion of healthy populations may have contributed to a floor effect, wherein smaller improvements were noted because there was less room to improve. This review did not include healthy participants, and although a meta-analytic synthesis of effect sizes was not conducted, small to moderate effect sizes were still reported most often. Spijkerman et al [25] also considered that poorer adherence to Web-based MBIs may contribute to lower effect sizes than face-to-face interventions, as Web-based interventions provide more anonymity and result in less accountability. They described adherence rates varying from 35% to 92% (which also varied somewhat because of differing definitions of adherence among different studies). Likewise, this study reported the average number of intervention sessions completed to range from 60% to 94%, and the proportion of those who participated in every session ranged from 30% to 69%. Given that regular practice is considered a necessity for development of mindfulness skills, and dose-response relationships have been found between amount of time practicing and degree of improvement [50], poor adherence can hamper the effectiveness of Web-based MBIs. It could also be that the relative absence of social support typical of Web-based interventions may partially account for the smaller effect sizes observed in outcomes related to psychological well-being such as anxiety and depressive symptoms.

Among the interventions that included mindfulness training as the core of the program [30,33-35,41-43,45,47], most primary outcomes improved over time and approximately half improved more in the intervention than control group. Among the interventions that included mindfulness as one component of a multimodal intervention [29,31,36,38-40,43], all primary outcomes improved over time and most improved more in the intervention than control group. However, the studies of interventions with mindfulness as a component of a multimodal intervention had over twice as many primary outcomes, were all asynchronous, and were all guided (whereas approximately half of the studies with mindfulness as the core of the intervention were synchronous or guided). Furthermore, primary outcomes were highly variable. Thus, it is difficult to determine to what extent the emphasis on mindfulness training alone in an intervention may have influenced observed improvements.

The results of this review give no clear indication of whether synchronous versus asynchronous Web-based MBIs or facilitated versus self-directed Web-based MBIs are more effective. Theoretically, synchronous and facilitated group interventions include more potentially therapeutic components such as social support and most closely align with traditional face-to-face program delivery. Furthermore, the presence of a facilitator could improve treatment adherence by ensuring that the participants actually understand and engage in the therapeutic processes, and they may feel more accountable to attend and practice at home. However, because of the small number of studies included in this review and relative heterogeneity of the specific interventions and outcomes investigated, any conclusion regarding the effectiveness of synchronous versus asynchronous or facilitated versus self-directed therapies would be premature.

### Limitations

There are some limitations that necessitate consideration. First, only published studies were included; thus, it is possible that results may be biased in favor of positive trial results. Second, only a small number of studies were included. Although patterns of results emerged, more cumulative findings need to be analyzed before definitive conclusions can be drawn, particularly among physical outcomes. Furthermore, although a population of those with a chronic physical condition is more homogeneous than mixed samples that have been reported in prior studies [25,51], there is still variability between people with physical health conditions, and thus different groups may be differently affected by MBIs. Analyses of results varying by condition are not possible with a small number of studies (especially when a specific population may only be represented in one study); thus, it is difficult to determine for which populations MBIs may be more or less effective. Third, as noted by Spijkerman et al [25], although all therapies included were unified by an emphasis on mindfulness, subtle differences exist between the therapies that may differentially affect outcomes. For example, there was differing relative emphasis on meditation practice, and some therapies included goal setting whereas others emphasized on nonstriving [25]. Finally, relevant studies may be missing from this review as studies not available in full-text or English language were excluded, and screening and data extraction were conducted by only 1 author.

### Future Work

As the delivery of Web-based MBIs for people with physical health conditions represents a new area of research, more high-quality studies are needed to establish the effectiveness of Web-based MBIs and to consider for whom they are most effective, for what outcomes, and using which specific MBIs. Whereas this review did not include studies where mindfulness was not the primary emphasis of the program, there are interventions with mindfulness components that appear to similarly benefit people with physical health conditions [52,53]. Studies comparing Web-based MBIs to active controls, as well as noninferiority studies directly comparing Web versions of MBIs with the face-to-face interventions on which they are based will be helpful in determining the utility of Web-based MBIs and whether they represent an equally effective delivery method. Adherence should also be consistently measured and described (eg, studies should report both the mean number of sessions completed and the proportion of participants who completed all sessions), and methods to improve adherence to Web-based MBIs should be investigated. Although all studies employed treatments in which mindfulness was a central component, few of the reviewed studies actually assessed changes in mindfulness levels from pre- to posttreatment. Future studies may consider including measures of mindfulness to investigate whether actual changes in dispositional mindfulness or mindfulness skills drive the observed improvements in symptoms. Finally, further research should examine whether synchronous interventions and the inclusion of therapist guidance (or the amount of guidance) result in greater improvement in outcomes.

### Conclusions

Web-based MBIs may be helpful for improving depressive symptoms, pain acceptance, fatigue, stress coping efficacy, family enjoyment, and social engagement. Furthermore, Web-based MBIs may be particularly effective when they are tailored for specific symptoms. Future studies should continue to compare Web-based MBIs to traditional delivery methods, and examine which features of Web-based MBIs are more or less effective for reduction of symptom burden.

## Appendix

Multimedia Appendix 1 Search strategy.

## Abbreviations

ACT: acceptance and commitment therapy
AIDS: acquired immunodeficiency syndrome
CBT: cognitive behavioral therapy
HRQoL: health-related quality of life
HIV: human immunodeficiency virus
IBS: irritable bowel syndrome
MBCPM: Mindfulness-Based Chronic Pain Management
MBCR: Mindfulness-Based Cancer Recovery
MBCT: Mindfulness-Based Cognitive Therapy
MBIs: Mindfulness-Based Interventions
MBSR: Mindfulness-Based Stress Reduction
MSER: Mindful Socioemotional Regulation
PRISMA: Preferred Reporting Items for Systematic Reviews and Meta-Analyses
NT-proBNP: N-terminal probrain natriuretic peptide
RCTs: randomized controlled trials
TAU: treatment as usual
