# Supplementary material for: Web-Based Mindfulness Interventions for People With Physical Health Conditions: Systematic Review
Source: J Med Internet Res. 2017 Aug 31;19(8):e303. doi: 10.2196/jmir.7487 (PMC5599726; doi:10.2196/jmir.7487)
Supplement: Multimedia Appendix 1 [file jmir_v19i8e303_app1.pdf]

Appendix A. PubMed Search Strategy

Search terms

1. Online
2. OR Internet
3. AND Mindfulness
4. AND Intervention

Search line: ((Online OR Internet) AND Mindfulness) AND Intervention
Field: All Fields
Dates: all dates included (search conducted November 2016)
Limits: none used
